# Nine-Second Cardiac Arrest in a Patient With Anti-mitochondrial Antibody-Positive Myopathy Under General Anesthesia

**DOI:** 10.7759/cureus.37436

**Published:** 2023-04-11

**Authors:** Atsuhiro Kitaura, Rina Yamamoto, Shota Tsukimoto, Shinichi Hamasaki, Yasuhumi Nakajima

**Affiliations:** 1 Anesthesiology, Kindai University, Osaka-Sayama, JPN

**Keywords:** myositis, anti-mitochondrial antibody, cardiac arrest, arrhythmia, bradycardia-tachycardia syndrome, general anesthesia, anti-mitochondrial antibody positive myositis, in hospital cardiac arrest

## Abstract

A small percentage of cases of dermatomyositis are positive for anti-mitochondrial antibodies (AMA), a known marker for primary biliary cirrhosis. AMA-positive myositis is a rare disease that has been reported to be accompanied by myocarditis, resulting in low left ventricular function, supraventricular arrhythmias, and abnormalities of the conduction system. We present a case of AMA-positive myocarditis resulting in sinus arrest during general anesthesia. A 66-year-old female with AMA-positive myocarditis underwent artificial femoral head replacement for osteonecrosis of the femoral head under general anesthesia. During general anesthesia, a nine-second sinus arrest occurred without any inducement. The sinus arrest was thought to be influenced by not only over-suppression caused by severe supraventricular tachycardia derived from sick sinus syndrome but sympathetic depression caused by general anesthesia. Because of the potential for life-threatening cardiovascular events during anesthesia in patients with AMA-positive myositis, it was considered essential to provide adequate preoperative management and intraoperative monitoring during anesthesia for patients with this disease. Herein, we report our case with a literature review.

## Introduction

Anti-mitochondrial antibodies (AMA) are characteristic of primary biliary cirrhosis (PBC) and are also implicated in myositis [1,2]. AMA-positive myositis is a rare autoimmune inflammatory condition that destroys muscle tissue and is frequently associated with myocardial dysfunction [1,2]. Myocardial dysfunction is characterized by left ventricular dysfunction and arrhythmias such as sinus failure, impaired conduction pathways, and supraventricular tachyarrhythmias [2]. Because of the potential for life-threatening cardiovascular events, perioperative management of patients with AMA-positive myositis might require attention. However, to our best knowledge, there are no reports of anesthetic experience in patients with AMA-positive myositis. Herein, we report a case of AMA-positive myocarditis resulting in sinus arrest during general anesthesia.

## Case presentation

A 66-year-old female was scheduled for bipolar hip arthroplasty for osteonecrosis of the femoral head. The patient had a history of AMA-positive myositis, paroxysmal atrial fibrillation (PAf), and chronic heart failure. She was taking 30 mg of edoxaban for PAf, which was to be continued during the perioperative period. Spinal block (epidural and spinal subarachnoid anesthesia) was not indicated; thus, anesthesia was planned to be performed with general anesthesia and peripheral nerve block.

Three years prior to the surgery, the patient was referred to our hospital by her primary doctor for symptoms of heart failure and atrial tachycardia and was admitted for heart failure. The cause of heart failure was examined and a diagnosis of AMA-positive myositis was made. Since that time, proximal muscle weakness, supraventricular arrhythmia, and left ventricular dysfunction had been observed, and a follow-up by a neurologist was started.

Five months prior to surgery, the patient was hospitalized for a second time for heart failure. Her left ventricular ejection fraction (LVEF) was 0.3 at that time. Heart failure due to myocarditis was suspected. Pending improvement of heart failure, steroid therapy (60 mg/day) was started for myositis. Magnetic resonance imaging revealed a high signal indicative of myositis on short tau inversion recovery imaging. Needle electromyography showed findings suggestive of myositis, such as resting potentials and some early recruitment, and findings suggesting possible myogenic changes in voluntary contraction. Muscle biopsy was also consistent with AMA myositis. Left ventricular myocardial biopsy showed fibrous degeneration with fat displacement and lymphocytic infiltration. As a result of treatment, creatine kinase was normalized and brain natriuretic peptide decreased; however, troponin I was persistently weakly positive. After confirming the efficacy of the treatment, the steroid dose was gradually tapered to 30 mg/day. Echocardiography performed after steroid therapy showed LVEF of 0.37 and mild mitral regurgitation. Her cardiac magnetic resonance revealed an ejection fraction of 0.31, global severe hypokinesis, and enlargement of both atria, but no significant late gadolinium enhancement. During hospitalization, beta-blocker was introduced for heart failure, PAf, and atrial tachycardia; however, it was discontinued due to sinus arrest that occurred twice after supraventricular tachycardia. Pacemaker implantation was suggested for pulse rate control, but the patient refused. As a result, control of the patient’s arrhythmia was poor, and the patient had paroxysmal atrial tachycardia or PAf for about eight hours a day.

One week prior to surgery, the patient visited our orthopedic department with a chief complaint of pain in the right hip. She was diagnosed with osteonecrosis of the right femoral head, presumably due to steroid therapy. One day before surgery, the patient was admitted to our hospital for an artificial femoral head replacement. Electrocardiography (ECG) after admission revealed atrial fibrillation (Af) with a heart rate of about 130 bpm. Transthoracic echocardiography showed mild mitral regurgitation and decreased global left ventricular wall motion (Video 1). Her left ventricular ejection fraction was 47%. Chest X-ray revealed no cardiac enlargement or pleural effusion.

**Video 1:** The patient’s echocardiogram the day before surgery. Transthoracic echocardiography showed mild mitral regurgitation, moderate tricuspid regurgitation, and decreased global left ventricular wall motion.

**Figure 1:**
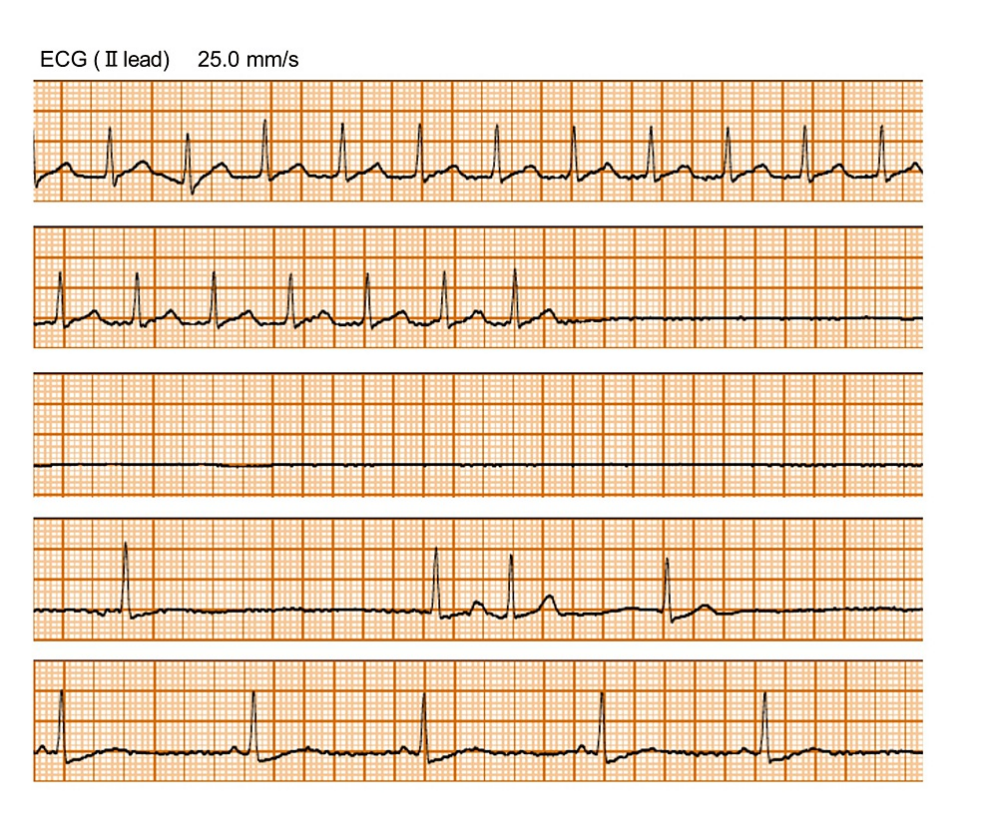
Electrocardiogram (Ⅱ lead) around the time of sinus arrest. Electrocardiogram revealed sinus arrest followed by atrial fibrillation. After the appearance of supplemental rhythm, the ECG returned to sinus rhythm.

On the day of surgery, a basic monitor was placed after admission to the operating room. ECG revealed sinus tachycardia (about 140 bpm). Intravenous catheterization was performed in the left forearm. After oxygenation, general anesthesia was induced with 2 mg/kg propofol, and a supraglottic device was inserted. After securing the airway, ultrasound-guided right femoral nerve and right lateral femoral cutaneous nerve blocks were performed with 20 mL of 0.3% ropivacaine. No arterial pressure line was secured. The patient was placed in the left lateral position for surgery. The patient was maintained intraoperatively with desflurane 5% and fentanyl (200 ug). Hydrocortisone 50 mg intravenously was administered for steroid coverage. Supraventricular tachycardia persisted after the start of anesthesia. About 60 minutes after the start of surgery, a sudden sinus arrest occurred (Figure 1). The anesthesiologist immediately called for backup and began preparing to administer adrenaline; however, the patient's pulse returned, and adrenaline was not administered. The duration of sinus arrest was about 9,000 ms. Post-recovery ECG showed a p-wave with narrow QRS (Figure 1), but the patient had persistent severe bradycardia. Therefore, 0.5 mg of atropine sulfate was administered. The p-wave gradually increased, but some atrioventricular block was still present. Immediately after improvement, the ECG was in sinus rhythm but quickly shifted to Af. Thereafter, the atrioventricular conduction disturbance gradually improved. During the course of the event, there were no ST changes in the ECG or changes in the partial pressure of carbon dioxide. She was managed by securing an arterial pressure line with an open arterial pressure line. The patient was awakened in the operating room and extubated. No similar events occurred in the operating room. After completion of the surgery, the patient was consulted by the arrhythmia department. She was suspected to have type 3 sinus failure (bradycardia-tachycardia syndrome). There was a need for intervention for tachycardia, and it was determined that pacemaker implantation was necessary to address sinus arrest caused by antiarrhythmic drugs.

## Discussion

AMA-positive myositis was first described in 1973 [1]. AMA-positive myositis is often preceded by systemic symptoms, such as muscle weakness, and is often associated with impaired conduction and cardiac function such as supraventricular arrhythmia, sinus failure, and atrioventricular block [2]. The mechanisms of these supraventricular arrhythmias and conduction defects remain unclear. Lymphocytic infiltrates and fatty fiber degeneration are common pathologic findings in myocarditis. There have been case reports of significant mitochondrial degeneration [3] and reports that impaired mitochondrial respiration in the myocardium [4] is involved in the development of cardiac diseases, such as cardiomyopathy; however, no evidence of myopathologic mitochondrial dysfunction in AMA cardiomyopathy has been demonstrated in large cohort studies to date [5]. AMA-positive myocarditis is more likely to result in diffuse myocardial damage than other cardiomyopathies [6]. As a result, the clinical presentation is more likely to include conduction defects such as sinusoidal failure and atrioventricular block. Inflammation and autonomic nervous system effects have also been suggested as mechanisms of tachyarrhythmia in myocarditis [7]. The autonomic nervous system is also involved in the development of sinus failure and Af [8].

The patient was recovering from an acute exacerbation of myocarditis, and the residual inflammation of myocarditis was prone to Af and sinus tachycardia. Inflammation and pain due to necrosis of the femoral head and intraoperative surgical invasion may have also affected the patient's condition. However, we discontinued beta-blockers because multiple short-duration sinus arrests were observed after their introduction. Ablation was not done based on the risk of atrioventricular block, and lack of heartbeat during sinus arrest (without the ectopic firing of atrial fibrillation, the patient might not be able to maintain a self-paced heartbeat during sinus arrest). Rate control by pacemaker was not performed due to a lack of patient consent. As a result, the present patient had inadequate preoperative control of supraventricular tachycardia.

The arrhythmic event was sinus arrest. While the ECG was flat, percutaneous arterial blood oxygen saturation was unmeasurable, pulse rate showed 0, and end-tidal carbon dioxide (EtCO2) was markedly decreased. A previous study reported that AMA myocarditis can be associated with sinus failure; however, the mechanism is not clear [5]. Spillover of myocarditis to the sinoatrial node may directly result in sinus failure; however, the extent to which the sinus node was compromised in the present patient is unknown, because no tissue sample was available. The present sinus event was preceded by severe supraventricular tachycardia, and a postoperative review of medical records of previous sinus arrest events revealed the same finding. Episodes of sinus arrest and preceding supraventricular tachycardia followed by temporary sinus rhythm after recovery of the self-paced heartbeat were characteristic. The patient's condition corresponded to type 3 sinus failure (bradycardia-tachycardia syndrome). Sinus arrest was thought to be due to overdrive suppression in response to severe supraventricular tachycardia [9]. To the best of our knowledge, the sinus arrest in the present study is the longest recorded (nine seconds), which may have been influenced by the suppression of the sympathetic nerves due to general anesthesia.

The appropriate response at the onset of sinus arrest was considered to be the same as for cardiac arrest. In the present case, the anesthesiologist immediately noticed sinus arrest; however, the duration of sinus arrest was short, and the patient's pulse was restored before adrenaline was administered. In addition, the patient was in a side-lying position, which made chest compressions difficult. Because patients with AMA myocarditis are at risk for sinus failure, atrioventricular block, and tachyarrhythmias, especially in cases of poor preoperative control, as in this case, monitoring should have taken into account the possibility of cardiac arrest. It is thereby important not to delay the initiation of intravenous adrenaline and chest compressions. In the present case, anesthesia management with an arterial pressure line and extracorporeal pacing on standby may have reduced the risk to the patient.

## Conclusions

We experienced an exceptionally long sinus arrest during general anesthesia in a patient with AMA-positive myositis. AMA-positive myositis can lead to arrhythmias such as sinus failure and supraventricular arrhythmias. In the anesthetic management of patients with AMA-positive myositis, careful investigation of their preoperative status and preparation for dangerous arrhythmias are important.
